# Rapid Accumulation of Polymorphonuclear Neutrophils in the *Corpus luteum* during Prostaglandin F_2α_-Induced Luteolysis in the Cow

**DOI:** 10.1371/journal.pone.0029054

**Published:** 2012-01-03

**Authors:** Koumei Shirasuna, Sineenard Jiemtaweeboon, Sybille Raddatz, Akane Nitta, Hans-Joachim Schuberth, Heinrich Bollwein, Takashi Shimizu, Akio Miyamoto

**Affiliations:** 1 Graduate School of Animal and Food Hygiene, Obihiro University of Agriculture and Veterinary Medicine, Obihiro, Japan; 2 Clinic for Cattle, University of Veterinary Medicine Hannover, Hannover, Germany; 3 Institute of Immunology, University of Veterinary Medicine Hannover, Hannover, Germany; Institut Jacques Monod, France

## Abstract

Prostaglandin F_2α_ (PGF_2α_) induces luteolysis within a few days in cows, and immune cells increase in number in the regressing *corpus luteum* (CL), implying that luteolysis is an inflammatory-like immune response. We investigated the rapid change in polymorphonuclear neutrophil (PMN) numbers in response to PGF_2α_ administration as the first cells recruited to inflammatory sites, together with mRNA of interleukin-8 (IL-8: neutrophil chemoattractant) and P-selectin (leukocyte adhesion molecule) in the bovine CL. CLs were collected by ovariectomy at various times after PGF_2α_ injection. The number of PMNs was increased at 5 min after PGF_2α_ administration, whereas IL-8 and P-selectin mRNA increased at 30 min and 2 h, respectively. PGF_2α_ directly stimulated P-selectin protein expression at 5–30 min in luteal endothelial cells (LECs). Moreover, PGF_2α_ enhanced PMN adhesion to LECs, and this enhancement by PGF_2α_ was inhibited by anti-P-selectin antibody, suggesting that P-selectin expression by PGF_2α_ is crucial in PMN migration. In conclusion, PGF_2α_ rapidly induces the accumulation of PMNs into the bovine CL at 5 min and enhances PMN adhesion *via* P-selectin expression in LECs. It is suggested that luteolytic cascade by PGF_2α_ may involve an acute inflammatory-like response due to rapidly infiltrated PMNs.

## Introduction

The bovine corpus luteum (CL) secretes progesterone (P) to establish and maintain pregnancy. If pregnancy is not established, the CL regresses with rapid loss of the ability to secrete P (functional regression) followed by disruption of vascular vessels and luteal cells (structural regression) [1], [2]. Luteolysis is caused by prostaglandin F_2α_ (PGF_2α_) secreted from the endometrium around days 17–19 of the estrous cycle or when exogenously given during the mid-luteal phase in the cow.

Various types of immune cells such as neutrophils, eosinophils, macrophages, and CD4-positive and CD8-positive T lymphocytes exist in the bovine CL and have essential roles in ovarian function [3], [4], [5]. During luteolysis, leukocytes, especially eosinophils, macrophages and T lymphocytes, are significantly increased in number, and 70% of all proliferating cells in the bovine CL are CD14-positive macrophages [5], [6]. Moreover, inflammatory cytokines such as tumor necrosis factor α (TNFα), interleukin 1β (IL-1β), and interferon γ (IFNγ), and chemokines such as monocyte chemoattractant protein-1 (MCP-1; recruitment of macrophages) are involved in luteal regression in cows [7], [8], [9], [10]. On the other hand, luteal cells express both class I and class II major histocompatibility complex (MHC) molecules (MHC molecules are essential to the recognition of cells by T lymphocytes as either self or non-self) [11], [12] and MHC class II expression on luteal cells is significantly increased when luteal regression is induced by PGF_2α_ [13], indicating that immune response occur between luteal cells expressed MHC class II and increased macrophages and T lymphocytes in the regressing CL. Benyo et al, [13] suggested that the demise of the CL may be involved in local autoimmune response mechanisms facilitated by increased expression of class II MHC molecules at the time of luteolysis. Additionally, the CL regresses primarily through the loss of cells by apoptosis [14], [15]. These findings suggest that the luteolytic phenomenon is an inflammatory-like immune response [3], [4], [5].

In the ovary, neutrophils are detected during the life span of the CL, and it is well known that neutrophils and its major chemoattractant, interleukin-8 (IL-8) is important for ovarian function [16], [17], [18], [19]. The recruitment of neutrophils implies overlapping succession of continuous events encompassing neutrophil inducement, rolling, and firm adhesion onto endothelial cells [20], [21]. On endothelial cells, P-selectin and E-selectin interact with neutrophils to promote their rolling and transient adhesion [22]. Other critical endothelial cell adhesion molecules, intercellular adhesion molecule (ICAM) and vascular cell adhesion molecule (VCAM) also mediate firm adhesion [23]. Generally, inflammation can be either acute or chronic [20], and acute inflammation is characterized by the infiltration of neutrophils, occurs within minutes, and dissipates within a few days [20].

We hypothesized that the bovine luteolysis involves an “acute” inflammatory-like immune response characterized by massive recruitment of neutrophils within the CL, consequently triggering a local immune response in the regressing tissue. In the present study, we investigated the number of polymorphonuclear neutrophils (PMNs) and mRNA expression of PMN migration-related factors at the early stage of PGF_2α_-induced luteolysis, and further examined to clarify the mechanisms of the rapid PMN migration by PGF_2α_ into the bovine CL.

## Results

### Number of PMNs in the bovine CL during PGF_2α_-induced luteolysis

**Figure 1:**
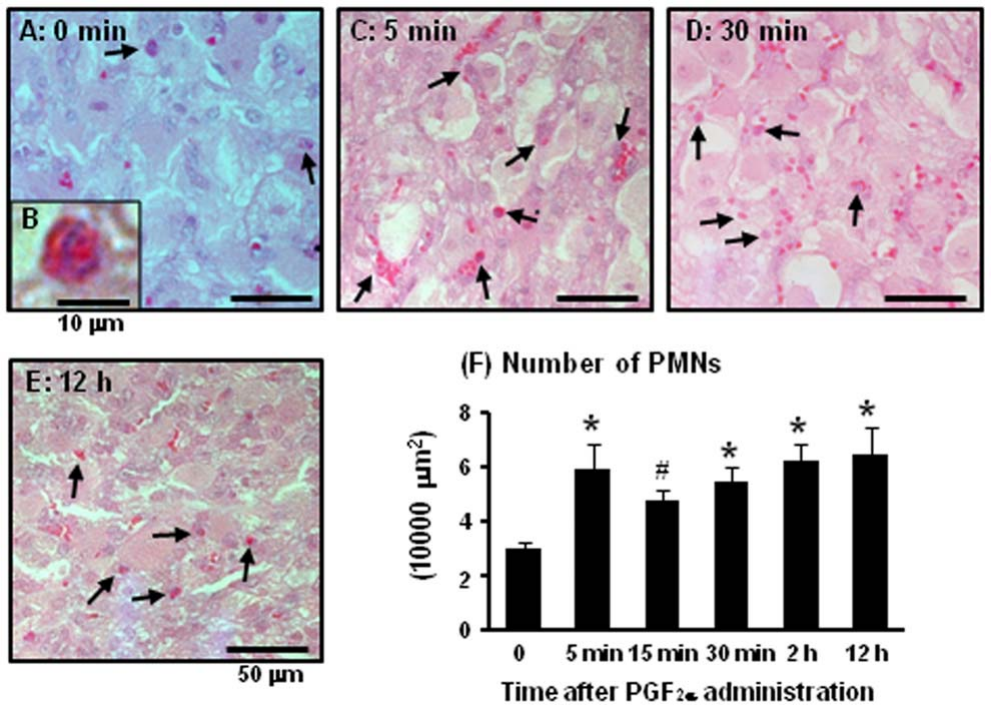
PMN numbers in the bovine CL during PGF_2α_-induced luteolysis. The typical images of PMNs within the CL at 0 min (Fig. 1A), 5 min (Fig. 1C), 30 min (Fig. 1D), and 12 h (Fig. 1E) during PGF_2α_-induced luteolysis. Fig. 1B indicates extended figure of PMN within the CL and Fig. 1F shows number of PMNs during PGF_2α_-induced luteolysis (n = 4−5 in each time), respectively. Black arrows show PMNs in the CL. Values are shown as the means ± SEM. Different superscripts indicate significant differences (P<0.05) as determined by ANOVA followed by Bonferroni's multiple comparison test.

Fig. 1 show PMNs within the CL at 0 min (Fig. 1A), 5 min (Fig. 1C), 30 min (Fig. 1D) and 12 h (Fig. 1E) after PGF_2α_ injection as detected by periodic acid-Schiff (PAS) staining. Fig. 1B indicate magnified figure of PMN which has 2–5 lobes of nuclear and finely-granular. The change in PMN number during PGF_2α_-induced luteolysis is shown in Fig. 1F. The number of PMNs within the CL was significantly increased (P<0.05) at 5 min, 30 min, 2 h, and 12 h after PGF_2α_ injection, and tended to increase at 15 min (P<0.1).

### Change of IL-8, P-selectin, E-selectin, ICAM and VCAM mRNA expression in the bovine CL and plasma P concentration during PGF_2α_-induced luteolysis

**Figure 2:**
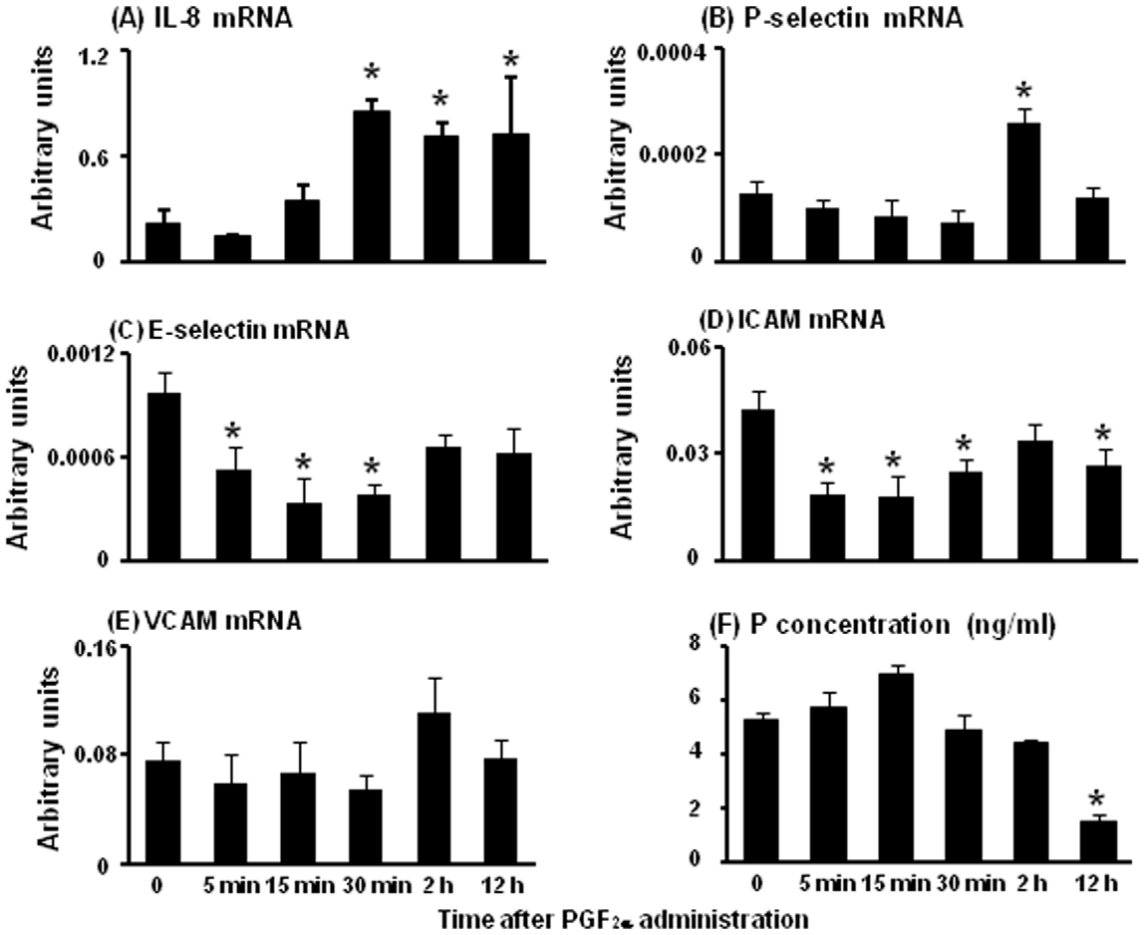
mRNA expression of IL-8, P-selectin, E-selectin, ICAM and VCAM in the bovine CL and plasma P concentration during PGF_2α_-induced luteolysis. Fig. 2 indicates the change of mRNA expression of IL-8, P-selectin, E-selectin, ICAM and VCAM within the CL and plasma P concentration at 0 min, 5 min, 15 min, 30 min, 2 h and 12 h during PGF_2α_-induced luteolysis (n = 4−5 in each time), respectively. Values are shown as the means ± SEM. Different superscripts indicate significant differences (P<0.05) as determined by ANOVA followed by Bonferroni's multiple comparison test.

IL-8 mRNA levels were significantly increased at 30 min, 2 h and 12 h after PGF_2α_ injection compared with 0 min (Fig. 2A). P-selectin mRNA levels were increased at 2 h after PGF_2α_ injection (Fig. 2B). E-selectin mRNA levels were significantly decreased at 5 min, 15 min and 30 min after PGF_2α_ injection compared with 0 min (Fig. 2C). ICAM mRNA levels were significantly decreased from 5 min to 30 min and 12 h after PGF_2α_ injection compared with 0 min (Fig. 2D) whereas VCAM mRNA expression did not change during PGF_2α_-induced luteolysis (Fig. 2E). The plasma P concentration did not differ at 5 min, 15 min, 30 min, and 2 h after PGF_2α_ injection compared with 0 min but significantly decreased at 12 h after PGF_2α_ injection (Fig. 2F).

### mRNA expression of IL-8 and P-selectin in luteal cells (LCs) and luteal endothelial cells (LECs)

**Figure 3:**
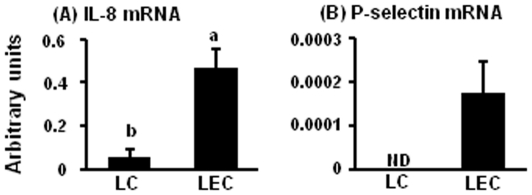
Expression of IL-8 and P-selectin mRNA in LCs and LECs. Fig. 3 indicates mRNA expression of IL-8 (A) and P-selectin (B) in LCs and LECs (n = 5 in each group). All values are the means ± SEM. Different superscript indicate significant differences (P<0.05) as determined by Student's *t*-test.

Based on the results of Fig. 2, we investigated mRNA expression of IL-8 and P-selectin in LCs and LECs. The mRNA of IL-8 (Fig. 3A) and P-selectin (Fig. 3B) in LCs and LECs are shown in Fig. 3. IL-8 mRNA expression was higher in LECs compared with LCs. P-selectin mRNA expressed only LECs but not in LCs.

### Effect of PGF_2α_ for mRNA expression of IL-8 and P-selectin in LECs and LCs

**Figure 4:**
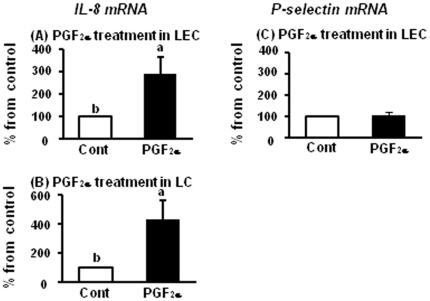
Regulation on mRNA expression of IL-8 and P-selectin by PGF_2α_. Fig. 4 indicates mRNA expression of IL-8 and P-selectin after PGF_2α_ (10^−6^ M) treatment for 2 h in LECs and LCs (n = 5 in each group). All values are the means ± SEM. Different superscript indicate significant differences (P<0.05) as determined by Student's *t*-test.

Based on the results of Fig. 3, we investigated mRNA expression of IL-8 and P-selectin by PGF_2α_ treatment in LECs and LCs. PGF_2α_ treatment for 2 h increased IL-8 mRNA expression both in LECs and LCs (Fig. 4A and 4B) compared with those in control, but PGF_2α_ treatment for 2 h did not effect on P-selectin mRNA expression in LECs (Fig. 4C).

### Acute effect of PGF_2α_ for P-selectin expression in LECs

**Figure 5:**
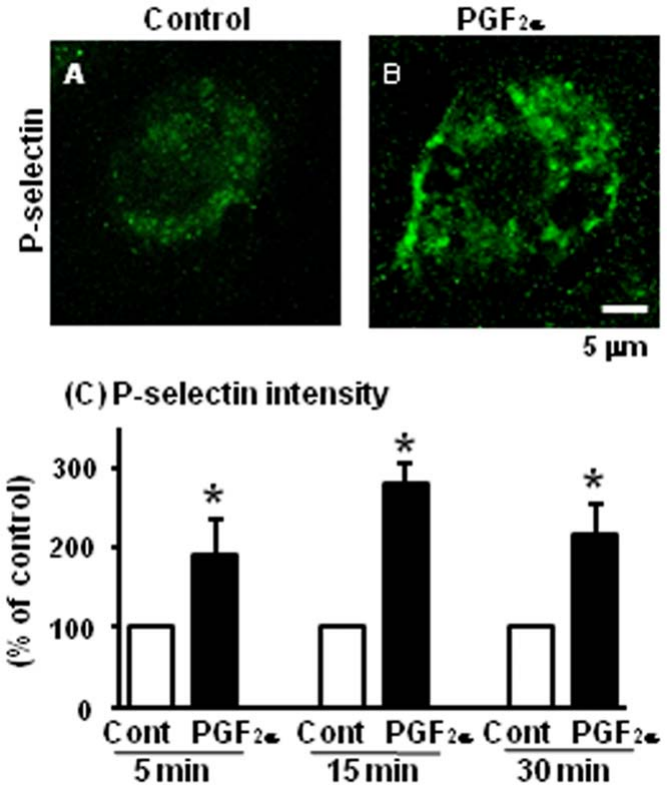
Effect of PGF_2α_ on P-selectin in LECs. The levels of P-selectin expression after PGF_2α_ (10^−6^ M) treatment in LECs. Fig. 5A shows P-selectin expression in LECs in control and Fig. 5B shows P-selectin expression in the PGF_2α_ treatment group at 5 min (n = 4 in each group). Scale bars indicate 5 µm. Fig. 5C shows the intensity of P-selectin expression at 5, 15 and 30 after treatment, respectively (% of control). All values are the means ± SEM. Different superscript indicate significant differences (P<0.05) as determined by Student's *t*-test.

We focused on the effect of PGF_2α_ for P-selectin protein expression in LECs since P-selectin is constitutively stored and the stimulation of endothelial cells by various mediators such as TNFα, can induce P-selectin translocation to cell surface membranes [24], [25], [26]. Fig. 5 displays typical images of P-selectin protein expression at 5 min in control (Fig. 5A) and after PGF_2α_ treatment (Fig. 5B) and P-selectin intensity (Fig. 5C) in LECs. PGF_2α_ treatment increased P-selectin expression (Fig. 5B) and its intensity (Fig. 5C) at 5, 15 and 30 min compared with those in control in LECs. We and other group previously confirmed that LECs used in the present study has PGF_2α_ receptor *in vitro* and *in vivo* [27], [28].

### Involvement of PGF_2α_ and P-selectin in the adhesion of PMNs to LECs

**Figure 6:**
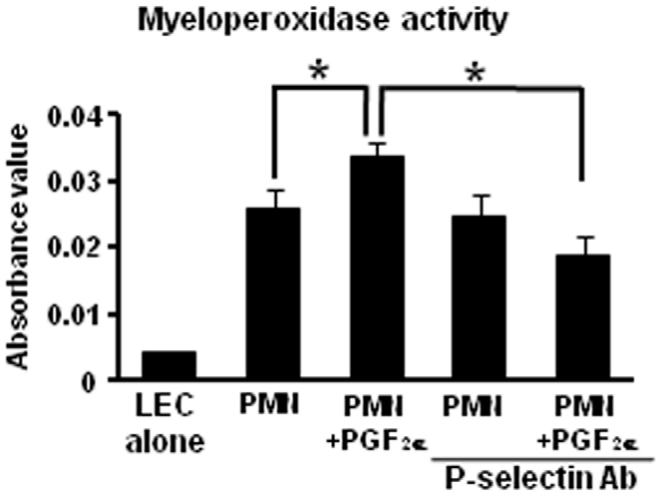
Effect of PGF_2α_ and P-selectin for PMN adhesion to LECs. The myeloperoxidase activity of PMNs was measured as an indicator of the number of PMNs (Fig. 6). Before the addition of PMNs, LECs were preincubated with or without anti-P-selectin (10 µg/mL) monoclonal antibody for 30 min. PMNs were added onto LECs with or without PGF_2α_ (10^−6^ M) for 30 min (n = 4 in each group). All values are the means ± SEM. Different superscripts indicate significant differences (P<0.05).

We investigated the impact of PGF_2α_ and P-selectin for adhesion of PMNs to LECs (Fig. 6). Before the addition of PMNs, LECs were preincubated with or without anti-P-selectin (10 µg/mL) antibody for 30 min. PMNs were added to the cultures in the presence or absence of PGF_2α_ (10^−6^ M) for 30 min because it took time for depression of PMNs on LECs. As shown in Fig. 6, the adhesion between PMNs and LECs was significantly enhanced by treatment with PGF_2α_ as compared with untreated PMNs (without PGF_2α_). This enhancement by PGF_2α_ of PMN adhesion to LECs was inhibited by pretreatment of LECs with anti-P-selectin antibody.

### Expression of FPr in PMNs, PBMCs, and the mid CL and PMN migration stimulated by PGF_2α_ and IL-8

**Figure 7:**
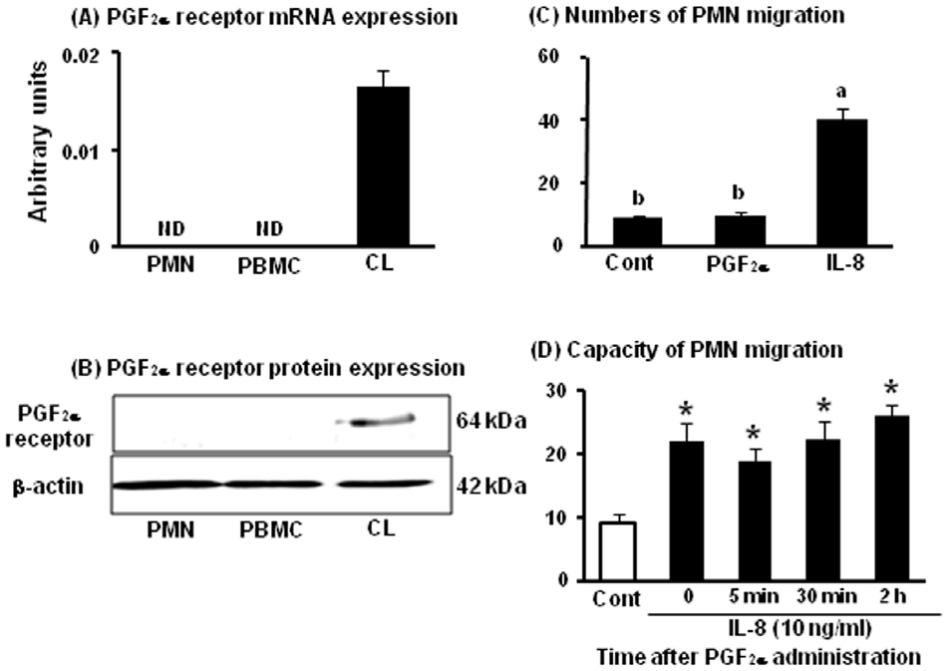
Expression of FPr in PMNs, PBMCs, and the mid CL and transmigration assay of PMNs by PGF_2α_ and IL-8. The mRNA and protein levels of FPr (A and B) in PMNs, PBMCs and the mid CL tissue (n = 4 in each group). All values are the means ± SEM (relative to β-actin mRNA levels). Representative photograph of a western blot is shown for FPr and β-actin (B). The number of PMNs migration by PGF_2α_ (10^−6^ M) and IL-8 (50 ng/ml) is shown in Fig 7C (n = 4 in each group). The number of migrating PMNs after PGF_2α_ administration is shown in Fig 7D. White bar indicates control (PMNs before PGF_2α_ administration, IL-8 un-stimulate) and black bars indicate IL-8 (10 ng/ml)-stimulated PMNs (at 0 min, 5 min, 30 min, and 2 h after PGF_2α_ administration, n = 4 in each time point). Values are shown as the means ± SEM. Different superscript indicate significant differences (P<0.05) as determined by ANOVA followed by the Bonferroni's multiple comparison test.

We further investigated the possibility whether PGF_2α_ directly stimulates the recruitment of neutrophils. The mRNA (Fig. 7A) and protein (Fig. 7B) expression of PGF_2α_ receptor (FPr) in PMNs, PBMCs, and the mid CL tissue are shown in Fig. 7. Liptak et al. reported that the bovine immune cells did not express FPr mRNA [29]. Same with the previous study, both FPr mRNA and protein were detected in the mid CL tissue but not in PMNs or PBMCs. The number of migrating PMNs in the transmigration assay is shown in Fig. 7C. IL-8 (50 ng/ml) significantly increased PMN migration about 4-fold compared to control. However, PGF_2α_ treatment (10^−6^ M) did not stimulate PMN migration. These findings suggest that PGF_2α_ cannot induce the PMN migration directly.

### Capacity of PMN migration during PGF_2α_-induced luteolysis

The number of migrating PMNs during PGF_2α_-induced luteolysis is shown in Fig. 7D. PMNs collected from before and at 5 min, 30 min, and 2 h after PGF_2α_ administration were used for the transmigration assay. IL-8 (10 ng/ml of IL-8 is significantly effective dose to stimulate PMN migration (data not shown)) stimulated PMN migration in all of the experimental groups; however, the levels of PMN migration did not change after PGF_2α_ administration relative to the levels before administration, suggesting that PGF_2α_ injection did not change the capacity/sensitivity of PMN migration.

## Discussion

In the present study, we hypothesized that the bovine luteolysis involves an “acute” inflammatory-like immune response characterized by massive recruitment of neutrophils within the CL. Regarding neutrophilic chemoattractants, mRNA expression of IL-8 started to increase at 30 min after PGF_2α_ administration. Additionally, P-selectin mRNA expression was increased at 2 h after PGF_2α_ administration. Surprisingly, the number of PMNs already increased at 5 min after PGF_2α_ administration before the mRNA levels of IL-8 and P-selectin increased, suggesting that PGF_2α_ injection may change the microenvironment of the CL to induce acute PMN accumulation in cows.

To clarify the mechanism of rapid PMN accumulation into the CL after PGF_2α_ injection, we examined whether PGF_2α_ affects adhesion system such as the rolling of immune cells *via* P-selectin. P-selectin mRNA was expressed in LECs but not in LCs (Fig. 3B) and increased at 2 h, but not earlier after PGF_2α_ injection *in vivo* (Fig. 2B). Other PMN adhesion-related factors such as E-selectin, ICAM and VCAM did not increase during the experimental period *in vivo*. In the *in vitro* experiment in the present study, we showed that PGF_2α_ treatment directly stimulated P-selectin expression at 5, 15 and 30 min in LECs (Fig. 5). Moreover, PGF_2α_ enhanced PMN adhesion to LECs, and this enhancement by PGF_2α_ of PMN adhesion was inhibited by anti-P-selectin antibody (Fig. 6). These findings suggest that P-selectin expression stimulated by PGF_2α_ is a key event in PMN migration within the CL. It has been shown that P-selectin is constitutively stored within Weibel-Palade bodies (WPBs) and the stimulation of endothelial cells by various mediators such as TNFα, vascular endothelial growth factor, angiopoietins, histamine, and thrombin can induce the fusion of WPBs to cell surface membranes and P-selectin translocation [24], [25], [26]. Moreover, the expression of P-selectin peaked within 5 min after thrombin challenge, and neutrophil adhesion induced by thrombin was inhibited by P-selectin antibody in human endothelial cells [24]. These findings imply that a rapid induction of P-selectin is an essential process in tissue inflammation, and the bovine CL may have a similar system to induce an acute inflammatory-like response during PGF_2α_-induced luteolysis.

In addition, we examined the expression of IL-8 and the effect of PGF_2α_ treatment in LCs and LECs *in vitro*. IL-8 mRNA was detected both in LCs and LECs. Actually, ovarian cells such as theca, granulosa and granulosa-lutein cells express IL-8 mRNA and produce IL-8 in cows [30], rabbits [31], rats [19] and human [16], [32]. In the *in vitro* cell culture experiment, PGF_2α_ treatment for 2 h stimulated IL-8 mRNA expression both in LCs and LECs. Indeed, in endometrial adenocarcinoma cells transfected with the FPr, PGF_2α_ promoted the synthesis and release of IL-8 in a time-dependent manner [33]. Additionally, the number of PMNs within the CL maintained high levels up to 12 h after PGF_2α_ administration, suggesting that PGF_2α_ may partly regulate the inflammatory microenvironment in the regressing CL *via* neutrophilic chemoattractant IL-8.

We further investigated the possibility whether PGF_2α_ directly stimulates the recruitment of PMNs into the CL. As Liptak et al. already showed that the bovine immune cells had no FPr mRNA [29], PMNs as well as PBMCs did not express PGF_2α_ receptor mRNA and protein (Fig. 7). Moreover, PGF_2α_ injection *in vivo* did not change the response level of PMN migration stimulated by IL-8 (Fig. 7D), suggesting that PGF_2α_ injection did not change the capacity/sensitivity of PMN migration. Thus, PGF_2α_ cannot directly attract immune cells into the CL, and the rapid and local induction such as P-selectin and IL-8 by PGF_2α_ within the CL is the key component to induce PMN migration in cows.

Neutrophils are the first cells recruited to inflammatory sites, providing cytokines and proteolytic enzymes [21]. In rats, co-incubation of luteal cells with activated neutrophils by *N*-formyl-methionyl-leucyl-phenylalanine (fMLP) reduced LH-stimulated cAMP accumulation and P secretion, which was dependent upon the number of neutrophils [34]. Moreover, a pretreatment with antibody against CD18 (leukocyte integrin) significantly inhibited not only PGF_2α_-induced neutrophil accumulation but also the decrease in serum P concentrations [35]. Also, neutrophils accumulate in the equine CL after PGF_2α_ administration [18]. Therefore, neutrophils infiltrated within the CL may also play a role in functional regression of the CL.

In the earliest phenomenon of luteolysis, oxytocin secretion in plasma from ovarian vein started to increase within 5 min after PGF_2α_ administration [36]. We also found in the present study the rapid increase of PMNs within the CL examined at 5 min after PGF_2α_ injection. Indeed, PGF_2α_ can induce PMN increase following the peritoneum injection in rats [37]. Generally, an acute inflammation is characterized by the infiltration of PMNs within a few minutes and continuous occurrence of lymphocyte and macrophage migration. PMNs can produce various types of inflammatory cytokines recruiting lymphocytes and macrophages such as IL-8, TNFα and IFNγ[21], [38], [39], [40]. Also, a large number of lymphocytes and macrophages were observed within the bovine CL at 6–24 h after PGF_2α_ administration, and these immune cells are considered to be essential for a rapid demise of the CL tissue [3], [4], [13], [41]. Indeed, inflammatory cytokines recruiting lymphocytes and macrophages such as TNFα and IFNγ clearly stimulate apoptosis in the bovine luteal cells and luteal endothelial cells [10], [42]. These findings suggest that acutely migrated PMNs have the potential to recruit other immune cells during luteolysis to progress structural regression in cows.

The mechanism of luteolysis is different depending on the species among cows, rodents and humans [43]. For example, although immune cells are also recruited into the CL to induce apoptosis and phagocytosis during luteolysis in rodents and humans same as cows, the initial signal of luteolytic mechanism seems to be different between these animals; as PGF_2α_ is the trigger of the bovine luteolysis, preovulatory prolactin surges are the trigger in cycling rats whereas absence of human chorionic gonadotropin could be a signal for initiation of luteolysis in humans [43]. Further investigation is required to determine whether the rapid accumulation of PMNs into the CL by the luteolytic trigger is the bovine specific phenomenon or common phenomenon between species.

In conclusion, PGF_2α_ rapidly induced the accumulation of PMNs within the bovine CL at 5 min after administration. PGF_2α_ treatment directly stimulated P-selectin expression and enhanced PMN adhesion in LECs *via* P-selectin. It is suggested that luteolytic cascade by PGF_2α_ involves an acute inflammatory-like response due to acute migrated PMNs in cows, and these PMNs may have a potential to recruit other immune cells in the regressing CL.

## Materials and Methods

The collection of CL in the experiment of PGF_2α_-induced luteolysis was conducted at the Clinic for Cattle, University of Veterinary Medicine Hannover, Germany. The experimental procedures complied with guidelines of the ethics committee on animal right protection of Oldenburg, Germany in accordance with German legislation on animal rights and welfare and the protocol was approved by the committee on the Ethics of Animal Experiments of University of Veterinary Medicine Hannover (Permit number: 33.9-42502-04-07/1275). The blood collection experiments were conducted at the Field Center of Animal Science and Agriculture, Obihiro University, and all experimental procedures complied with the Guidelines for the Care and Use of Agricultural Animals of Obihiro University and the protocol was approved by the committee on the Ethics of Animal Experiments of Obihiro University (Permit number: 19–10).

### *In vivo* study: PGF_2α_-induced luteolysis

For collecting the CL during luteolysis, 29 normal cyclic German Holstein cows were used for this study. The day of estrus was designated as day 0. Cows (n = 4−5 for each time point) at the mid luteal phase (days 10–12) were injected with PGF_2α_ in intramuscular (0 h) (0.5 mg of cloprostenol, 2.0 mL of Estrumate™, Essex Tierarznei, Munich, Germany), and ovaries were collected by ovariectomy [44] through the vagina at 0 ( = before PGF_2α_ injection), 5, 15, and 30 min and 2 and 12 h. Simultaneous plasma samples were collected by caudal venipuncture for P measurements, and were immediately frozen at −20°C until further analysis.

### Processing of the *corpus luteum*

The CL was enucleated from the ovary and dissected free of connective tissue as previously described [28]. Subsequently, the CL was fixed with 10% formaldehyde and embedded in paraffin wax according to the standard histological technique. In addition, the remaining CL was prepared for molecular biological purposes. The CL tissue samples were collected, minced, immediately placed into a 1.5 mL microcentrifuge tube with or without 0.4 mL of TRIzol reagent (Invitrogen, Karlsruhe, Germany), and stored at −80°C until analysis.

### *In vitro study:* Expression and regulation of IL-8 and P-selectin mRNA in steroidogenic luteal cells and luteal endothelial cells by PGF_2α_

#### Steroidogenic luteal cells (LCs) culture

The CLs of the mid luteal phase were collected at local slaughterhouse, and dispersed using collagenase IV (SIGMA, St. Louis, MO, USA). The luteal stages were classified as mid (days 10–12) by macroscopic observation of the ovary as described previously [45]. LCs were used in the present study using the method described by Klipper et al. [46]. Briefly, LCs were isolated from the bovine mid CL (Days 8–12 of the estrous cycle) using magnetic tosylactivated beads coating with BS-1 lectin (binds glycoproteins on the bovine endothelial cells), indicating BS-1 positive cells are endothelial cells. In the present study, BS-1 negative cells were assessed as LCs as described previously [46]. LCs were cultured in DMEM/F-12 medium (Invitrogen Corporation, Tokyo, Japan) containing 5% fetal bovine serum (FBS; Invitrogen Corporation), 2.2% NaHCO3, gentamicin solution (50 mg/L, SIGMA), and amphotericin B solution (2.5 mg/L, SIGMA).

#### Luteal endothelial cells (LECs) culture

LECs were used in the present study using the method described by Spanel-Borowski et al. [47]. Cytokeratin-negative LECs isolated from the CLs of the cows during the mid-luteal phase were used as described previously [47]. LECs were grown on plates pre-coated with 1% Vitrogen in DMEM/F-12 medium containing 5% FBS, 2.2% NaHCO3, gentamicin solution, and amphotericin B solution. All experiments in the present study were carried out on LECs from passages 5 to 8.

For analysis of mRNA expression, LCs and LECs were cultured for 24 h at 37°C, were placed into a 1.5 mL microcentrifuge tube with 400 µL of TRIzol reagent and stored at −80°C until analysis.

#### Treatment with PGF_2α_

LCs were cultured for 24 h after isolation and LECs were grown until confluent, rinsed with PBS twice, and stimulated with medium only (control) or PGF_2α_ (10^−6^ M) for 2 h at 37°C in DMEM/F-12 medium containing 0.1% FBS, NaHCO3, gentamicin solution, and amphotericin B solution. At the end of the treatment period, cells were collected and stored at −80°C until the mRNA expression was analyzed. At least 5 experiments were performed, with each concentration of agents tested with 2 replicates/experiments.

### Effect of PGF_2α_ on P-selectin expression in LECs

#### Treatment with PGF_2α_ and immunofluorescence

The experimental method was modified from that of Maliba et al. [26]. LECs were grown until confluent, rinsed with PBS twice, and stimulated with PGF_2α_ (10^−6^ M, SIGMA) for 5, 15 and 30 min in DMEM/F-12 medium containing 0.1% FBS, 2.2% NaHCO3, gentamicin solution, and amphotericin B solution. LECs were fixed with 1% paraformaldehyde-PBS solution for 20 min at room temperature. Following fixation, the cells were washed with PBS 3 times and incubated with blocking solution (4% normal goat serum in PBS) for 15 min at room temperature. Cells were incubated with mouse anti-human P-selectin reacted with cow antibody (1∶100 dilution, AbD serotec) for 24 h at 4°C. Cells were rinsed with PBS and incubated with rabbit anti-mouse conjugated to Alexa 488 IgG (1∶400 dilution, Invitrogen) for 60 min at room temperature. The cells were observed with a confocal microscope (DMI6000B, Leica Microsystems, USA), and rabbit anti-mouse conjugated to Alexa 488 was visualized using a 488-nm argon laser. Intensity of P-selectin staining was calculated by pixel sum/area of LECs using accessory software for this microscopy. In each experiment, at least 20 LECs were calculated the intensity of P-selectin. At least 4 experiments were performed, with each concentration of agents tested with 2 replicates/experiments.

### Effect of PGF_2α_ and anti-P-selectin antibody for PMN adhesion to LECs

#### Isolation of PMNs and PBMC

PMNs and peripheral blood mononuclear cells (PBMCs) were isolated from whole blood collected via jugular veinipuncture on days 8–12 of the estrous cycle (day of ovulation  = day 1) as described previous our study [48]. Blood samples were centrifuged at 1000×g for 30 min at 10°C over Lymphoprep^TM^ (Axis-Shield, Oslo, Norway) according to the manufacture's method. The plasma was removed and buffy coat were separated as PBMCs, and lower layer under buffy coat used for separation of PMNs. To remove red blood cells, hypotonic distilled water was added to PMNs and PBMCs for approximately 5 s. Isotonicity was restored by the addition of twice-concentrated PBS and centrifuged at 500×g for 10 min at 10°C. This lysis procedure was repeated twice on the cell pellet. Isolated PMNs and PBMCs were resuspended at a concentration of 2×10^6^ cell/mL in RPMI 1640 (Invitrogen Corporation) containing 0.1% FBS, gentamicin solution, and amphotericin B solution. To check the purity of PMNs before using for the experiment, the purity of PMNs was >95% and these cells resulted in nearly pure granulocyte populations as determined by flow cytometric evaluation (Beckman Coulter, Inc., CA, UAS) [48]. Additionally, we observed giemsa-stained PMNs by microscope, these cells were clear granule and segmented nuclear. Although peripheral granulocytes include neutrophils, eosinophils and basophils in generally, the purity of neutrophils (2–5 lobes of nuclear and finely-granular) was >95% in these PMNs by microscope observation in the present study since character was different between neutrophils and eosinophils (double nuclear and coarsely-granular). Moreover, in the flow cytometric evaluation of PBMCs, the purity of PBMCs was >96% assessed by lower granular and these cell size [48].

#### PMNs-LECs adhesion assay

The experimental method was modified from that of Yasuda et al [49]. LECs were grown until confluent. Before the addition of PMNs, LECs were preincubated with or without anti-P-selectin (10 µg/mL) monoclonal antibody for 30 min. PMNs isolated from blood sample (n = 4) during the mid-luteal phase (1×10^6^ cell/mL) were added to the cultures in the presence or absence of PGF_2α_ (10^−6^ M) for 30 min in DMEM/F-12 medium containing 5% FBS, NaHCO3, gentamicin solution, and amphotericin B solution. After incubation, non-attached cells were washed out 3 times with PBS containing 2% FBS, and 100 µL of citrate buffer (SIGMA) containing 0.1% Triton X-100 was added to each well. After 10 min, 200 µL of *o*-phenylenediamine dihydrochloride (SIGMA)-citrate buffer solution containing 0.04% H_2_O_2_ was added to each well. After incubation at room temperature for 30 min, 50 µL of 4N-H_2_SO_4_ was added to stop the reaction, and the myeloperoxidase activity of neutrophils was measured at OD_490_. At least 4 experiments were performed, with each concentration of agents tested with 2 replications/experiments.

### PGF_2α_ receptor (FPr) mRNA and protein expression

#### Sample collection

PMNs and PBMCs were isolated from whole blood on days 10–12 of the estrous cycle (n = 4) as described above. Ovaries with the CL from Holstein cows were collected at a local slaughterhouse, and the mid CL were collected as described above (n = 4). For analysis of mRNA and protein expression, PMNs, PBMCs, and CL tissues were placed into a 1.5 mL microcentrifuge tube with or without 400 µL of TRIzol reagent and stored at −80°C until analysis.

### Transmigration assay of PMNs by PGF_2α_ and IL-8

#### Transmigration assay

PMN chemotaxis was evaluated using a 10-well microchemotaxis chamber (Neuro probe, Gaithersburg, MD, USA). In this instrument, test solutions in the bottom chamber are separated from leukocytes in the upper chamber by an 8- µm pore size filter (Neuro probe). The lower well was pre-filled with 100 µL of 100% isotonic Percoll (Pharmacia, Freiburg, Germany) to avoid adherence and loss of transmigrated PMNs to the bottom and wall of the lower well. The following solutions (300 µL) were pipetted onto the bottom chamber: (1) RPMI 1640 medium alone as a control, (2) PGF_2α_ (10^−6^ M; SIGMA), and (3) 50 ng/ml of the recombinant bovine IL-8 (Kingfisher, Biotech, Inc. St. Paul, MN, USA) as a positive control. Fifty ng/ml of IL-8 is significantly maximal does to stimulate neutrophil migration (data not shown). After assembling the instrument, PMNs were added to the upper chamber (250 µL/well, 2×10^6^ cell/mL). After incubation at 37 °C in 5% CO_2_ for 3 h, migrated cells in the bottom chamber were counted under light microscopy. At least 4 experiments were performed, with each concentration of agents tested with 2 replications/experiments.

### Transmigration assay of PMNs before and after PGF_2α_ administration

#### Blood sample collection

For collecting PMNs, 4 normal cyclic non-lactating Holstein cows were used for this study. The day of estrus was designated as day 0. Cows (n = 4 for each time point) at the mid luteal phase (days 10–12) were injected with PGF_2α_ (0 h) and blood samples were collected before and 5 min, 30 min, and 2 h after PGF_2α_ administration. Then, PMNs were separated as described above and set concentrations as 2×10^6^ cell/mL in RPMI 1640 medium.

#### Transmigration assay

The following solutions and PMNs (250 µL/well, 2×10^6^ cell/mL) were pipetted onto the bottom and upper chamber: (1) RPMI 1640 medium in the bottom and neutrophils (before PGF_2α_ injection) in the upper chamber as a control and (2) 10 ng/ml of IL-8 in the bottom and PMNs (at 0 min, 5 min, 30 min, and 2 h after PGF_2α_ injection) in the upper chamber as experimental groups. Ten ng/ml of IL-8 is significantly effective does to stimulate PMN migration (data not shown). After incubation at 37 °C in 5% CO_2_ for 3 h, migrated cells in the bottom chamber were counted under light microscopy.

### P determination

The plasma P concentration was determined by direct enzyme immunoassays (EIA) [45]. The minimum detectable concentration of the assay was 0.3 ng/mL. The intra- and interassay coefficients of variation were 6.2% and 12.5%, respectively. P was extracted using diethyl ether as described previously [45]. The recovery rate of P was 88%. The intra- and interassay coefficients of variation were 6.2% and 9.3%, respectively.

### Detection of neutrophils in the CL using periodic acid-Schiff (PAS) reaction

Formalin-fixed sections (5 µm) of the CL samples were stained with PAS reagent (Sigma) for 10 min and then counterstained with hematoxylin described as previous study [18], [48]. In general, it is recognized that PAS staining is useful method to detect granulophilic leukocytes. Although red blood cells and other immune cells such as macrophages are stained in the CL tissue, therefore in addition to positive cells by PAS staining, we checked the shape of nuclear of cells and assessed segmented granulocytes as PMNs. Thus, we could distinguish PMNs especially neutrophils from other PAS stained positive cells such as red blood cells and macrophages. This PAS stain was used on each tissue block, and 5 fields per section were examined at x 400 magnification. Quantification of the number of PMNs was performed independently by 3 observers. The results were expressed as means ± SEM per unit area.

### RNA extraction and cDNA production

Total RNA was extracted from leukocytes following the protocol of Chomczynski and Sacchi with TRIzol reagent [50] as described in our previous study [51]. The extracted total RNA was stored in RNA storage solution (Ambion, Texas, USA) at –80°C until being used for cDNA production. RNA samples were then used to produce cDNA as described in our previous study [51]. The synthesized cDNA was stored at −30°C.

### Quantitative real-time reverse transcription-polymerase chain reaction

Quantification of mRNA expression for IL-8, P-selectin, E-selectin, ICAM, FPr and β-actin was performed using synthesized cDNA via real-time PCR with a LightCycler (Roche Diagnostics Corp. Mannheim, Germany) with a commercial kit (QuantiTect^TM^ SYBR Green PCR, QIAGEN GmbH, Hilden, Germany). The amplification program consisted of 15 min of activation at 95°C followed by 40 cycles of PCR steps (15 sec of denaturation at 94°C, 30 sec of annealing at 60°C, and 20 sec of extension at 72°C). For quantification of the target genes, a series of standards was constructed by amplifying a fragment of DNA (150–250 bp) that contains the target sequence for real-time PCR. The primers used for real-time PCR were as follows: 5′-cctcttgttcaatatgacttcca-3′, forward, and 5′- ggcccactctcaataactctc-3′, reverse for IL-8 (Accession No. NM_173925); 5′-gccacctaggaacatacggagtt-3′, forward, and 5′-gattggacgaggtcaccaaga-3′, reverse for P-selectin (Accession No. NM_174183.2); 5′- actcccttggcagttggactt-3′, forward, and 5′-aggcgtttcagaagccagaa-3′, reverse for E-selectin (Accession No. NM_174181); 5′-ctctgtccatgggattctgaca-3′, forward, and 5′-gtttcatgtgaccctgtggtgtag-3′, reverse for ICAM (Accession No. NM_174348); 5′-ttggatggtgtttgcagtttct-3′, forward, and 5′-agtcagtgaaacagagtcaccaatct-3′, reverse for VCAM (Accession No. NM_001101158.1); 5′-tcagccctcacccagataagt-3′, forward, and 5′-ggccatttcactgttcagg-3′, reverse for FPr (Accession No. NM_181025.2); and 5′-ccaaggccaaccgtgagaaaat-3′, forward,and5′-ccacattccgtgaggatcttca-3′, reverse forβ-actin (Accession No. NM_173979.3). The values were normalized using β-actin as the internal standard.

### Western blotting

Detection of FPr in PMNs, PBMCs, and the mid CL were analyzed as described in our previous study [28]. We used polyclonal antibodies for FPr (64 kDa, Cayman Chemical, Michigan, USA. 1∶1000 dilution) an anti-β-actin antibody (42 kDa, SIGMA, 1∶5000 dilution), and horseradish peroxidase (HRP)-conjugated anti-rabbit (GE Healthcare, 1∶5000 dilution) and anti-mouse IgG antibodies (Rockland Immunochemicals, Inc. USA, 1∶10000 dilution). The signals were detected using an ECL Western Blotting Detection System (GE Healthcare UK Ltd., Little Chalfont, UK). The optical density of the immunospecific bands was quantified by means of an imageJ computer-assisted analysis system.

### Statistical analysis

All data are presented as means ± SEM. The statistical significance of differences was assessed by one-way ANOVA followed by Bonferroni's multiple comparison test or Student's *t*-test. Probabilities less than 5% (P<0.05) were considered significant.
